# Systemic immune-inflammation index: a key biomarker guiding personalized adjuvant chemotherapy in intrahepatic cholangiocarcinoma

**DOI:** 10.3389/fonc.2025.1702336

**Published:** 2025-10-31

**Authors:** Xin Yin, Yanjiang Yin, Wenzhuo Li, Jinliang Tong, Yi Liu, Jianping Chang, Jindong Ma, Yaoyu Xie, Xin Li, Hossein Mehrabanidil, Xiao Chen, Yefan Zhang, Jianqiang Cai, Caiyun Li, Bowen Xu, Zhiyu Li, Xinyu Bi

**Affiliations:** ^1^ Department of Hepatobiliary Surgery, National Cancer Center/National Clinical Research Center for Cancer/Cancer Hospital, Chinese Academy of Medical Sciences and Peking Union Medical College, Beijing, China; ^2^ Cancer Center, Beijing Tsinghua Changgung Hospital, School of Clinical Medicine, Tsinghua Medicine, Tsinghua University, Beijing, China; ^3^ Beijing Key Laboratory of Cell and Gene Therapy for Digestive System Cancers, National Cancer Center/National Clinical Research Center for Cancer/Cancer Hospital, Chinese Academy of Medical Sciences and Peking Union Medical College, Beijing, China; ^4^ Department of Gynecological Oncology, National Cancer Center/National Clinical Research Center for Cancer/Cancer Hospital & Shenzhen Hospital, Chinese Academy of Medical Sciences and Peking Union Medical College, Shenzhen, Guangdong, China; ^5^ Cheeloo College of Medicine, Shandong University, Jinan, China; ^6^ Department of Hepatobiliary Surgery, General Surgery, Cheeloo College of Medicine, Qilu Hospital, Shandong University, Jinan, China

**Keywords:** intrahepatic cholangiocarcinoma, systemic immune-inflammation index, adjuvant chemotherapy, prognosis, biomarker

## Abstract

**Background:**

Intrahepatic cholangiocarcinoma (ICC) is a highly aggressive primary liver malignancy, with poor long-term outcomes even after curative-intent resection. Postoperative adjuvant chemotherapy (pAC) is increasingly used, but its benefit is not uniform across all patients. The systemic immune-inflammation index (SII) has emerged as a potential prognostic marker in several cancers, but its role in ICC remains unclear.

**Methods:**

We retrospectively analyzed 445 ICC patients who underwent R0 hepatic resection at a single tertiary center between 2000 and 2023. Preoperative SII was calculated, and patients were stratified into high- and low-SII groups. The impact of SII on overall survival (OS) and recurrence-free survival (RFS) was evaluated, along with its interaction with pAC. Multivariate Cox regression models and maximally selected rank statistics were used for analysis.

**Results:**

The median follow-up was 34.3 months. High SII independently predicted worse OS and RFS (p < 0.001), outperforming conventional inflammatory and nodal indices. Lymph node ratio (LNR) also independently predicted survival but did not modify the effect of pAC. Interaction analysis revealed that pAC significantly improved OS in high-SII patients (5-year OS: 33% with pAC vs. 23% without; HR 0.62, 95% CI 0.42–0.94, p = 0.022) but conferred no significant benefit in low-SII patients (5-year OS: 49% with pAC vs. 55% without; HR 0.71, 95% CI 0.48–1.05, p = 0.089).

**Conclusions:**

SII is a robust prognostic biomarker in ICC and can guide individualized postoperative therapy. High-SII patients derive substantial survival benefit from adjuvant chemotherapy, whereas low-SII patients may be spared unnecessary treatment. Integrating SII into postoperative risk stratification may optimize outcomes and reduce overtreatment in ICC.

## 1 Introduction

Intrahepatic cholangiocarcinoma (ICC) is a malignant tumor originating from the epithelial cells of the intrahepatic bile ducts, ranking as the second most common type of primary liver cancer (1, 2). Its incidence has risen dramatically worldwide over the past decades, particularly in East and Southeast Asia, where risk factors such as hepatolithiasis, viral hepatitis, and metabolic disorders are prevalent (3, 4). Surgical resection remains the cornerstone and only potentially curative treatment for ICC (5, 6). Advances in hepatic surgery and perioperative management have expanded the pool of patients eligible for resection and improved short-term outcomes (7–9). Nevertheless, the long-term prognosis remains unsatisfactory, with 5-year survival rates after curative-intent resection ranging only from 20% to 40% (5). High incidence of recurrence and distant metastasis after resection underscore the need for effective postoperative strategies.

Adjuvant chemotherapy has emerged as a critical approach to reduce recurrence risk and improve survival in ICC patients, but the role of postoperative adjuvant chemotherapy (pAC) has long been debated (10, 11). Several retrospective studies and meta-analyses suggest that adjuvant therapy can improve outcomes in resected ICC, particularly in patients with high-risk features such as node-positive or margin-positive disease (12–16). Nonetheless, prospective evidence remains limited. Randomized trials specifically in ICC are lacking due to the tumor’s relative rarity and trial design challenges. Instead, most data on adjuvant therapy for ICC has been extrapolated from trials that enrolled heterogeneous biliary tract cancers (including perihilar cholangiocarcinoma and gallbladder cancer). Notably, results from these trials have been inconsistent. The UK BILCAP trial and the Japanese ASCOT trial demonstrated a survival benefit of adjuvant therapy (capecitabine and S-1, respectively) in biliary cancers (17–19). In contrast, other phase III trials such as the Japanese BCAT (gemcitabine monotherapy) and French PRODIGE 12 (gemcitabine-oxaliplatin) failed to show a significant overall survival advantage for adjuvant chemotherapy (20, 21). Given these mixed outcomes, the universal application of pAC for ICC remains controversial. There is a pressing clinical need to better stratify ICC patients to determine who is most likely to benefit from adjuvant therapy. Ideally, such risk stratification would spare low-risk patients the toxicity and cost of unnecessary chemotherapy, while ensuring high-risk patients receive additional treatment to improve their prognosis.

Accumulating evidence indicates that inflammatory response and immune status are pivotal factors influencing cancer progression and treatment outcomes (22, 23). Markers of systemic immune-inflammation have emerged as significant prognostic indicators in ICC, such as neutrophil-to-lymphocyte ratio (NLR), platelet-to-lymphocyte ratio (PLR), Glasgow prognostic score (GPS), and prognostic nutritional index (PNI) (24–27). The systemic immune-inflammation index (SII), a composite immune-inflammatory biomarker, has been associated with poor prognosis in various cancers (28–32). Elevated SII reflects a state of relative neutrophilia and thrombocytosis accompanied by relative lymphopenia, indicating alterations in inflammatory-immune status that are closely linked to tumor progression. As SII can be calculated from routine hematological tests, it offers non-invasiveness and high accessibility, underscoring its potential clinical utility. However, its prognostic significance in ICC and potential role in guiding therapeutic strategies remain insufficiently explored.

In this study, we conducted a large retrospective cohort study to investigate the prognostic value of SII in ICC patients who underwent surgery. In addition, we evaluated whether the SII was superior in predicting survival of ICC patients when compared with other inflammatory indices. Furthermore, we performed an interaction analysis to determine whether SII modifies the effect of adjuvant chemotherapy on patient outcomes. This study aims to develop a risk-adapted framework for personalized adjuvant therapy in ICC, thereby improving survival while avoiding overtreatment.

## 2 Methods

### 2.1 Data source and patient selection

We conducted a retrospective study of patients with ICC who underwent surgery at the Cancer Hospital, Chinese Academy of Medical Sciences (CHCAMS). Eligible patients were those who received curative-intent liver resection for pathologically confirmed ICC between January 2000 and March 2023. Curative-intent resection was defined as complete macroscopic tumor removal with histologically negative margins (R0 resection). Patients were excluded if postoperative pathology demonstrated distant metastasis (M1 disease), an R2 resection (macroscopically positive margin), or if essential clinicopathological data were unavailable. The study protocol was approved by the institutional review board of CHCAMS. After applying inclusion and exclusion criteria, 445 patients were deemed eligible for analysis.

Adjuvant treatment status was documented. Adjuvant chemotherapy (AC) was defined as the administration of at least one cycle of systemic chemotherapy after surgery according to institutional protocols. At our center, the decision to initiate AC was determined by a multidisciplinary tumor board, taking into account performance status and pathological risk factors. In general, AC was recommended for patients with high-risk features, including nodal metastasis, microvascular invasion, perineural invasion, poor differentiation, or tumor size >5 cm. Common regimens during the study period included gemcitabine/cisplatin doublet therapy and fluoropyrimidine-based therapy (capecitabine or S-1), in line with evolving standards of care. Patients who did not receive systemic therapy were categorized as the observation (non-AC) group. No patients in this cohort received adjuvant radiotherapy.

### 2.2 Variables and outcomes

Demographic and clinical variables included age, sex, year of surgery, presence of cirrhosis, use of neoadjuvant therapy, and extent of liver resection (major hepatectomy involving ≥3 segments vs. minor hepatectomy <3 segments). Tumor characteristics were obtained from pathology reports and included maximum tumor diameter, tumor number (solitary vs. multiple intrahepatic lesions), histologic grade, and adverse features such as microvascular invasion, perineural invasion, and invasion of adjacent organs or the liver capsule. Pathologic staging was assigned according to the 8th edition of the American Joint Committee on Cancer (AJCC) staging for ICC, including T category (T1–T4) and N category (N0: no nodal metastasis; N1: regional nodal metastasis). Lymphadenectomy of the porta hepatis was routinely performed. The total number of lymph nodes (LNs) retrieved and the number of metastatic LNs were recorded for each patient, and the lymph node ratio (LNR) was calculated as the number of metastatic LNs divided by the total number examined (range 0–1). By definition, LNR = 0 corresponded to N0 disease, whereas LNR > 0 indicated N1 disease.

Preoperative laboratory data obtained closest to the surgery date included complete blood counts, liver function tests, and tumor markers (carbohydrate antigen 19–9 [CA19-9] and carcinoembryonic antigen [CEA]). Several inflammation- and nutrition-related indices were calculated: SII = (neutrophils × platelets) ÷ lymphocytes (all ×10^9/L); C-reactive protein-Albumin-Lymphocyte (CALLY) index = albumin (g/L) × lymphocytes ÷ [C-reactive protein (mg/L) × 10]; and PNI = albumin (g/L) + 5 × lymphocytes (10^9/L). Serum CA19-9 > 37 U/mL and CEA > 5 ng/mL were defined as elevated, based on standard clinical cutoffs.

The primary endpoint was overall survival (OS), defined as the interval from surgery to death from any cause or last follow-up. The secondary endpoint was recurrence-free survival (RFS), defined as the interval from surgery to the first documented recurrence or progression, or to death in the absence of recurrence. Patients without events were censored at the last follow-up. All surviving patients were followed for at least 5 years or until study completion. Surveillance followed a standardized institutional protocol: physical examination, liver function tests, and tumor markers (CA19-9, CEA) every 3 months for the first 2 years, and every 6 months thereafter until 5 years. Imaging (contrast-enhanced abdominal computed tomography [CT] or magnetic resonance imaging [MRI] and chest imaging) was performed every 3–6 months or as clinically indicated.

### 2.3 Treatment and follow-up

All patients underwent curative-intent (R0) hepatic resection for ICC. The extent of resection was determined by tumor size and location, ranging from anatomic hemi-hepatectomy to segmentectomy or wedge resection for peripheral lesions. Extrahepatic bile duct resection or vascular resection/reconstruction was performed when necessary to achieve negative margins. Regional lymphadenectomy of the hepatoduodenal ligament was routinely performed. The decision to administer postoperative AC was made by a multidisciplinary team, with strong consideration for patients exhibiting high-risk pathological features. Adjuvant regimens evolved over the study period: most commonly gemcitabine-based chemotherapy (gemcitabine plus cisplatin, or gemcitabine alone in earlier years), or fluoropyrimidine-based therapy (capecitabine or S-1), depending on available evidence and drug accessibility.

Patients were closely monitored after surgery. Follow-up visits were scheduled at 1 month postoperatively, every 3 months for the first 2 years, and every 6 months thereafter up to 5 years. Each visit included clinical assessment, laboratory testing (including liver function, CA19-9, and CEA), and imaging as described. Recurrence was defined radiologically (new lesion consistent with cholangiocarcinoma) or pathologically if biopsy was performed. For relapsed patients, the date and site of first recurrence were recorded.

### 2.4 Statistical analysis

All analyses were performed using SPSS version 26.0 (IBM Corp.) and R version 4.0.3 (R Foundation for Statistical Computing). Continuous variables were expressed as mean ± standard deviation or median with interquartile range (IQR), as appropriate; categorical variables were summarized as frequencies and percentages. Baseline characteristics were compared between the AC and observation groups using Student’s t test or Mann–Whitney U test for continuous variables, and Chi-square or Fisher’s exact test for categorical variables, to assess potential selection biases.

Survival outcomes (OS and RFS) were estimated by the Kaplan–Meier method and compared using the log-rank test. Kaplan–Meier curves were stratified by key variables of interest, including receipt of AC (yes vs. no) and SII (high vs. low), to visualize survival differences. Prognostic factors were analyzed using Cox proportional hazards regression. Univariate analysis was first performed for each variable to calculate hazard ratios (HRs) and 95% confidence intervals (CIs). Variables with p < 0.05 in univariate analysis were entered into multivariable models using a backward stepwise elimination approach to identify independent predictors of OS and RFS. Proportional hazards assumptions were verified (e.g., using Schoenfeld residuals).

To evaluate whether SII modified the survival benefit of AC, we conducted interaction analyses. Separate multivariable Cox models for OS and RFS included an interaction term between AC (yes vs. no) and SII (modeled both as a continuous variable and dichotomized at an optimal cutoff). A statistically significant interaction term (p < 0.05) indicated that the effect of AC differed across SII levels. The optimal SII cutoff for OS discrimination was determined using the maximally selected rank statistic (MaxStat method), which identifies the threshold that maximizes standardized log-rank statistics. This was implemented using the surv_cutpoint function in the survminer package in R. Patients were then stratified into low- and high-SII groups, and survival outcomes were compared within these strata according to AC status using log-rank tests and Cox regression. A two-sided p < 0.05 was considered statistically significant.

## 3 Results

### 3.1 Baseline patient characteristics

A total of 445 patients with ICC met the inclusion criteria and underwent curative-intent hepatectomy during the study period. The clinicopathological characteristics are summarized in **Table 1**. The median age at surgery was 60 years (IQR, 52–64), and 59.6% were male. Underlying liver cirrhosis was present in 12.4% of patients. Histologically, most tumors were moderately differentiated (39.1%) or moderately-to-poorly/poorly differentiated (54.8%), whereas only 6.1% were well differentiated. Pathological staging according to the AJCC 8th edition showed that 52.4% of patients had stage II disease and 47.6% had stage III disease; no patients had stage I disease, consistent with the tertiary referral pattern, and none had stage IV disease per study criteria. The median tumor diameter was 4.5 cm (IQR, 3.4–6.5 cm), and 27.4% had multifocal tumors. Adverse features were frequent: 45.4% had microvascular invasion, 44.9% had perineural invasion, 19.8% had direct invasion of adjacent organs or liver capsule, and 13.0% harbored satellite nodules. Regional lymphadenectomy yielded a median of 6 lymph nodes (IQR, 4–10) per patient. Pathologic nodal metastasis was confirmed in 138 patients (31.0%), while 307 (69.0%) were node negative. Among node-positive cases, the number of nodes involved ranged from 1 to 21 (median, 2). The LNR varied widely, with an LNR ≥0.5 observed in 16.6% of node-positive patients.

**Table 1:** Baseline clinicopathologic characteristics of ICC patients.

Characteristics	non-pAC	pAC	P value
n	245	200	
Gender, n (%)			0.449
Female	103 (23.1%)	77 (17.3%)	
Male	142 (31.9%)	123 (27.6%)	
Initial treatment age, mean ± sd	60.229 ± 9.4802	58.66 ± 9.3182	0.081
Differentiation, n (%)			0.097
Moderate	125 (23.6%)	69 (15.5%)	
Poor and Moderate-Poor	100 (29%)	115 (25.8%)	
Moderate-Well and Well	20 (2.5%)	16 (3.6%)	
TNM stage, n (%)			0.368
Stage II	133 (29.9%)	100 (22.5%)	
Stage III	112 (25.2%)	100 (22.5%)	
Pathological T stage, n (%)			0.879
T2	160 (36%)	135 (30.3%)	
T3	79 (17.8%)	60 (13.5%)	
T4	6 (1.3%)	5 (1.1%)	
Pathological N stage, n (%)			0.008
N0	182 (40.9%)	125 (28.1%)	
N1	63 (14.2%)	75 (16.9%)	
CA19-9, median (IQR)	62.953 (18.21, 241.6)	53.009 (15.753, 512.55)	0.919
CEA, median (IQR)	2.48 (1.7, 3.63)	2.5 (1.61, 4.2725)	0.396
Diameter of tumor, median (IQR)	4.5 (3.4, 6.7)	4.5 (3.425, 6)	0.395
Tumor position, n (%)			0.891
Left liver lobe	107 (24%)	87 (19.6%)	
Right liver lobe	105 (23.6%)	83 (18.7%)	
Middle liver lobe	33 (7.4%)	30 (6.7%)	
Liver capsule invasion, n (%)			0.374
Yes	169 (38%)	130 (29.2%)	
No	76 (17.1%)	70 (15.7%)	
Satellite nodules, n (%)			0.045
No	206 (46.3%)	181 (40.7%)	
Yes	39 (8.8%)	19 (4.3%)	
Intravascular carcinoma embolus, n (%)			0.538
Yes	108 (24.3%)	94 (21.1%)	
No	137 (30.8%)	106 (23.8%)	
Perineural invasion, n (%)			0.001
Yes	93 (20.9%)	107 (24%)	
No	152 (34.2%)	93 (20.9%)	
Surrounding tissues Invasion, n (%)			0.001
Yes	62 (13.9%)	26 (5.8%)	
No	183 (41.1%)	174 (39.1%)	
Number of lymph node dissections, median (IQR)	5 (3, 9)	6 (4, 13)	0.001
Positive lymph node ratio, median (IQR)	0.33333 (0.2, 0.51316)	0.42262 (0.25, 0.6875)	0.028
Operation time, median (IQR)	220 (165, 274)	223 (180, 295.75)	0.275
Intraoperative hemorrhage, median (IQR)	200 (20, 400)	200 (20, 500)	0.681
Intraoperative blood transfusion, n (%)			0.976
Yes	37 (8.3%)	30 (6.7%)	
No	208 (46.7%)	170 (38.2%)	
Postoperative days, median (IQR)	8 (7, 12)	9 (8, 11)	0.976
SII, median (IQR)	654.74 (420.99, 854.87)	654.74 (445.84, 824.2)	0.636
CALLY, median (IQR)	1.2919 (0.45118, 6.3752)	2.0064 (0.56209, 7.8215)	0.106
PNI, median (IQR)	52.65 (47.95, 56.4)	52.35 (46.746, 56.05)	0.898

Preoperative serum tumor markers also demonstrated broad variability. The median CA19–9 level was 57.3 U/mL (IQR, 16.7–288.7), and 42% of patients had values above the upper limit of normal. The median CEA level was 2.48 ng/mL (IQR, 1.66–3.90), with 14% exceeding 5 ng/mL. Systemic inflammation and nutritional indices were heterogeneous: the median SII was 654.7 (IQR, 437.9–842.6), median PNI was 52.6 (IQR, 47.2–56.1), and median CALLY index was 1.67 (IQR, 0.47–7.56). Approximately 25% of patients had an elevated SII >850, whereas 25% had a low SII <420.

### 3.2 Baseline differences between AC and non-AC cohorts

Of the 445 patients, 200 (44.9%) received AC, while 245 (55.1%) did not. Treatment allocation was non-randomized and based on clinical judgment, resulting in baseline imbalances between groups (**Table 1**). As expected, patients in the AC group exhibited more high-risk features: lymph node metastasis was more frequent compared with the surgery-only group (37.5% vs. 25.7%, p = 0.008), and perineural invasion was also more common (53.5% vs. 38.0%, p = 0.001). Multiple tumors and satellite nodules were slightly more prevalent in the AC group, with satellite nodules showing borderline significance (15.9% vs. 9.5%, p = 0.045). In contrast, there were no significant differences between groups in age, sex, tumor size, preoperative CA19-9, or extent of hepatectomy (all p > 0.05). Importantly, preoperative inflammatory and nutritional indices (SII, CALLY index, PNI) were comparable between groups (e.g., median SII ~660 in both, p = 0.740), indicating that these parameters did not influence the decision for AC. This balance suggests that any survival differences observed by SII are unlikely to be explained by confounding in chemotherapy selection, but rather reflect the intrinsic prognostic or predictive value of SII.

### 3.3 Survival outcomes and univariate Cox analysis

By the censoring date, the median follow-up for the cohort was 34.3 months (95% CI, 32.9–36.0). During this period, 301 patients (67.6%) experienced tumor recurrence and 284 (63.8%) died. The Kaplan–Meier estimated OS rates at 1, 3, and 5 years after surgery were 84.2%, 52.3%, and 41.2%, respectively. The median OS was 38.4 months (95% CI, 32.9–59.3). Notably, 44 patients (9.9%) remained recurrence-free beyond 5 years, representing the long-term survivors in this series. RFS rates at 1, 3, and 5 years were 60.3%, 34.1%, and 24.8%, respectively, with a median RFS of 14.4 months (95% CI, 13.4–18.6). The steep decline between 1-year and 3-year RFS highlights the high rate of early recurrence characteristic of ICC.

**Figure 1:**
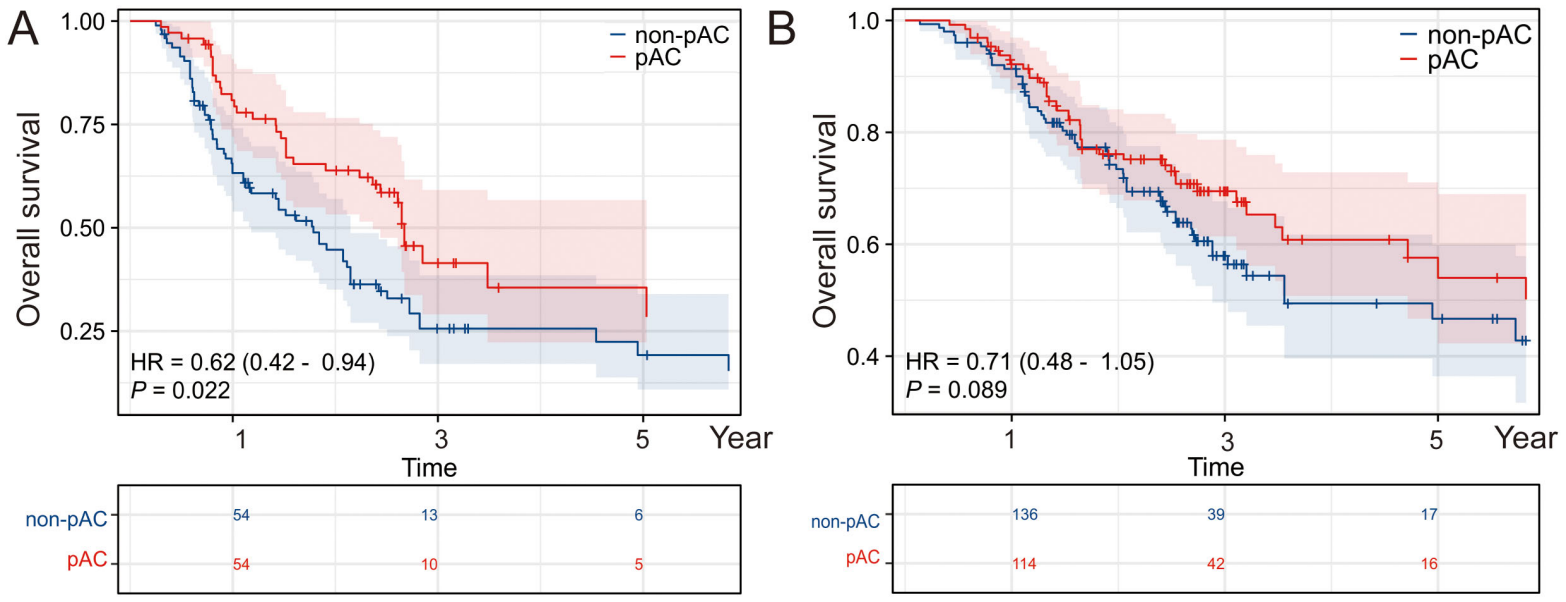
Impact of postoperative adjuvant chemotherapy on OS and RFS. Kaplan–Meier survival analyses depicting **(A)** overall survival (OS) and **(B)** recurrence-free survival (RFS) in patients who received postoperative adjuvant chemotherapy (pAC) compared with those who did not. Patients receiving pAC demonstrated improved survival outcomes, underscoring the potential therapeutic benefit of pAC in this cohort.

On univariate Cox analysis (**Table 2**), several factors were significantly associated with poorer OS: advanced AJCC stage (III vs. II, HR ≈2.33, p < 0.001), higher T category (T3/T4 vs. T1/T2, p < 0.001), lymph node metastasis (N1 vs. N0, HR ≈2.32, p < 0.001), elevated preoperative CA19–9 and CEA (both p < 0.001), capsular invasion (p = 0.021), microvascular invasion (HR ≈1.88, p < 0.001), perineural invasion (HR ≈1.33, p = 0.043), and extrahepatic extension (HR ≈1.44, p = 0.026). Longer operative time (p = 0.023), increased blood loss (p = 0.041), and prolonged hospital stay (p = 0.036) were also modestly associated with worse OS. Importantly, receipt of AC was associated with improved OS (HR = 0.710, 95% CI, 0.536–0.940; p = 0.017), corresponding to a ~29% reduction in mortality risk (**Figure 1A**).

**Table 2:** Univariate and multivariate Cox regression analyses of OS for ICC patients.

Characteristics	Total (N)	Univariate analysis	P value	Multivariate analysis	P value
		Hazard ratio (95% CI)		Hazard ratio (95% CI)	
Gender	445				
Female	180	Reference			
Male	265	1.145 (0.865 - 1.516)	0.343		
Initial treatment age	445	1.004 (0.990 - 1.018)	0.587		
Differentiation	445				
Moderate	174	Reference		Reference	
Poor and Moderate-Poor	244	1.339 (1.006 - 1.783)	0.045	1.173 (0.861 - 1.596)	0.312
Moderate-Well and Well	27	0.398 (0.161 - 0.984)	0.046	0.512 (0.198 - 1.321)	0.166
TNM stage	445				
Stage II	233	Reference		Reference	
Stage III	212	2.326 (1.757 - 3.080)	< 0.001	1.307 (0.836 - 2.045)	0.241
Pathological T stage	445				
T2	295	Reference		Reference	
T3	139	1.705 (1.278 - 2.273)	< 0.001	1.203 (0.807 - 1.793)	0.363
T4	11	3.473 (1.690 - 7.139)	< 0.001	2.516 (1.148 - 5.517)	0.021
Pathological N stage	445				
N0	307	Reference		Reference	
N1	138	2.316 (1.741 - 3.080)	< 0.001	0.940 (0.565 - 1.564)	0.811
CA 19-9	445	1.000 (1.000 - 1.000)	< 0.001	1.000 (1.000 - 1.000)	0.264
CEA	445	1.008 (1.005 - 1.011)	< 0.001	1.004 (0.998 - 1.010)	0.173
Diameter of tumor	445	1.028 (0.967 - 1.093)	0.379		
Postoperative adjuvant chemotherapy	445				
No	245	Reference		Reference	
Yes	200	0.710 (0.536 - 0.940)	0.017	0.635 (0.471 - 0.858)	0.003
Tumor position	445				
Left liver lobe	194	Reference			
Right liver lobe	188	0.827 (0.616 - 1.110)	0.206		
Middle liver lobe	63	0.759 (0.495 - 1.165)	0.208		
Liver capsule invasion	445				
Yes	299	Reference		Reference	
No	146	0.690 (0.504 - 0.945)	0.021	0.848 (0.604 - 1.191)	0.341
Satellite nodules	445				
No	387	Reference			
Yes	58	1.352 (0.930 - 1.967)	0.114		
Intravascular carcinoma embolus	445				
Yes	202	Reference		Reference	
No	243	0.531 (0.403 - 0.700)	< 0.001	0.627 (0.468 - 0.841)	0.002
Perineural invasion	445				
Yes	200	Reference		Reference	
No	245	0.754 (0.573 - 0.992)	0.043	0.981 (0.720 - 1.337)	0.905
Surrounding tissues Invasion	445				
Yes	88	Reference		Reference	
No	357	0.693 (0.502 - 0.956)	0.026	1.057 (0.736 - 1.518)	0.764
Number of lymph node dissections	445	1.005 (0.985 - 1.025)	0.608		
Proportion of positive lymph nodes	442	3.959 (2.612 - 5.999)	< 0.001	2.653 (1.184 - 5.947)	0.018
Operation time	445	1.002 (1.000 - 1.004)	0.023	1.002 (1.000 - 1.004)	0.018
Intraoperative hemorrhage	445	1.000 (1.000 - 1.001)	0.041	1.000 (1.000 - 1.000)	0.776
Intraoperative blood transfusion	445				
Yes	67	Reference			
No	378	0.868 (0.597 - 1.261)	0.458		
Postoperative days	445	1.032 (1.002 - 1.063)	0.036	1.008 (0.977 - 1.041)	0.617
SII	445	1.001 (1.000 - 1.001)	< 0.001	1.001 (1.000 - 1.001)	< 0.001
CALLY	445	1.001 (0.994 - 1.007)	0.840		
NLR	445	1.758 (0.914 - 2.747)	0.347		
PLR	445	1.578 (1.156 - 1.757)	0.034	1.187 (0.947 – 1.344)	0.374
PNI	445	1.006 (0.998 - 1.014)	0.132		

SII and LNR emerged as particularly strong predictors of survival. SII, analyzed as a continuous variable, had an HR of 1.001 per unit increase (p < 0.001). Although the per-unit effect was small, the wide distribution of SII values meant that an increase from the 25th percentile (~420) to the 75th percentile (~855) corresponded to an estimated 35% increase in mortality risk. Patients in the highest quartile of SII (>850) had significantly worse survival compared to those in the lowest quartile (<420) (log-rank p < 0.001). Similarly, LNR demonstrated a striking prognostic impact. Each 0.1 increment in LNR was associated with a significantly higher hazard of death. Progression from LNR = 0 (no positive nodes) to LNR = 1.0 (all nodes positive) conferred nearly a four-fold increased risk of death (HR 3.96, 95% CI, 2.61–5.99; p < 0.001). Intermediate values were also informative; for example, an LNR of 0.5 carried roughly twice the risk of an LNR of 0.1. These findings indicate that LNR provides more granular prognostic information than the conventional binary N0 vs. N1 classification, consistent with evidence from colorectal and gastric cancers.

A parallel analysis was conducted for RFS (**Supplementary Table S1**). Prognostic trends were broadly similar to OS. On univariate analysis, stage III, T3/T4, N1, elevated CA19–9 and CEA, larger tumor size, vascular invasion, perineural invasion, high SII, AC (**Figure 1B**), and high LNR were all associated with shorter RFS (all p < 0.05).

### 3.4 Multivariate Cox analysis

In multivariate Cox regression (**Table 2**), variables significant on univariate analysis and clinically relevant factors were included. After stepwise selection, SII remained an independent prognostic factor (p < 0.001). Despite adjustment for stage, nodal status, and treatment, higher SII continued to predict worse OS. Similarly, LNR retained independent prognostic value (adjusted HR = 2.65, 95% CI, 1.18–5.95; p = 0.018). Notably, once LNR was incorporated, the N category (N1 vs. N0) lost statistical significance, underscoring the superiority of LNR as a measure of nodal disease burden.

Adjuvant chemotherapy also remained an independent protective factor (adjusted HR = 0.635, 95% CI, 0.471–0.858; p = 0.003), corresponding to a 36% reduction in mortality risk. Tumor burden additionally influenced outcomes: T4 tumors were associated with significantly worse OS compared to T2 (HR = 2.516; p = 0.021), whereas T3 tumors did not differ significantly after adjustment. Microvascular invasion independently predicted worse OS (adjusted HR = 0.627 for absence vs. presence; p = 0.002). Other variables such as tumor size, multiplicity, or differentiation were not independently significant once SII and LNR were included, likely due to collinearity.

In multivariate analysis, tumor size independently predicted RFS (HR ≈1.08 per cm, p = 0.013), although it did not independently predict OS. This suggests that larger tumors increased recurrence risk, but did not significantly impact survival after recurrence, potentially reflecting limited salvage options. SII and LNR again remained independently prognostic (both p < 0.01), along with tumor size and vascular invasion. Adjuvant chemotherapy was associated with improved RFS, though the effect was less pronounced than for OS (adjusted HR ≈0.80; p = 0.07), suggesting AC may delay recurrence in some patients without consistently preventing it.

### 3.5 Interaction between SII and AC and survival outcomes by SII subgroup

We examined whether the effect of pAC on survival varied according to preoperative SII. In a multivariable Cox model including an interaction term (pAC × SII), a significant interaction was observed for OS (interaction HR = 0.95, 95% CI 0.91–1.00, p = 0.033), indicating that the survival benefit of pAC increased with higher SII. Other interactions were tested, with pathological T stage showing significance (HR 0.58, p = 0.014 for pAC × T3/T4), while LNR did not significantly interact with pAC, suggesting the benefit of chemotherapy was not markedly influenced by nodal burden when SII and T stage were accounted for (**Table 3**).

**Table 3:** Summary of each interaction tested with adjuvant chemotherapy in a multivariable individual Cox proportional hazards model.

Characteristics	Hazard ratio (95% CI)	P value
Differentiation	0.76 (0.39–1.45)	0.404
TNM stage	0.69 (0.38–1.29)	0.246
Pathological T stage	0.47 (0.25–0.90)	0.022
Pathological N stage	1.36 (0.71–2.60)	0.348
CA 19-9	1.00 (1.00 - 1.00)	0.417
CEA	1.01 (1.00–1.02)	0.072
Liver capsule invasion	0.81 (0.41–1.59)	0.534
Intravascular carcinoma embolus	1.21 (0.66–2.21)	0.543
Perineural invasion	1.28 (0.68–2.40)	0.444
Surrounding tissues Invasion	0.81 (0.36–1.82)	0.609
Number of lymph node dissections	2.22 (0.83–5.91)	0.111
Proportion of positive lymph nodes	0.78 (0.24–2.51)	0.673
Operation time	1.00 (1.00–1.00)	0.818
Intraoperative hemorrhage	1.00 (1.00–1.00)	0.526
Postoperative days	1.01 (0.94–1.07)	0.871
SII	1.001 (1.000–1.002)	0.013

**Figure 2:**
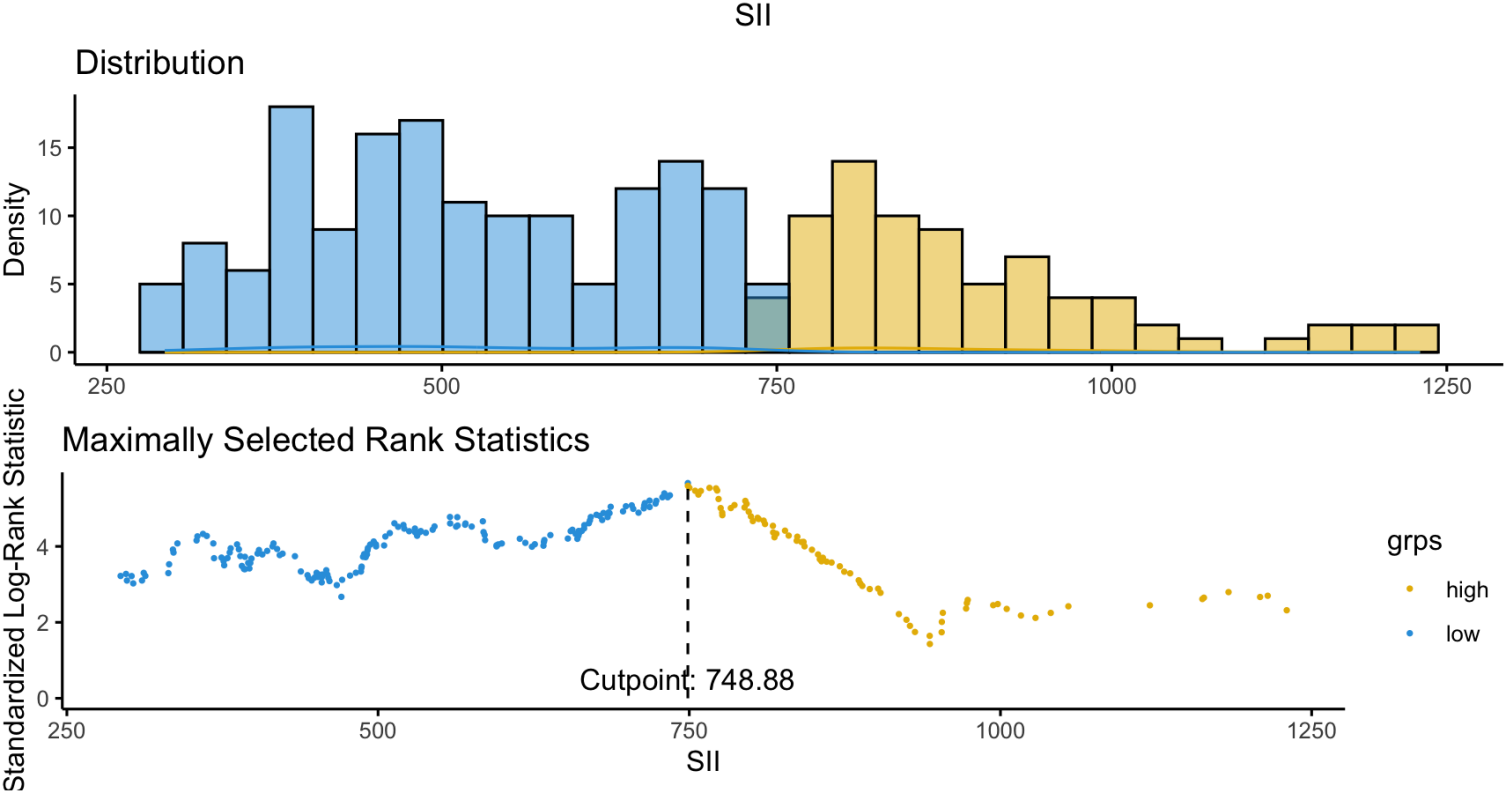
Optimal cutoff determination of SII for survival analysis. To further evaluate the interaction between SII and postoperative adjuvant chemotherapy (pAC), the MaxStat method was applied to identify the best threshold of SII for overall survival (OS) stratification. An SII value of 748.88 was determined as the optimal cutoff, effectively separating patients into two distinct prognostic subgroups (high SII vs. low SII).

To further explore this interaction, the maximally selected rank statistic (MaxStat) method identified an optimal SII cutoff of 748.88 for OS stratification (**Figure 2**). Patients were categorized as low SII (≤748.9; n = 267, 60.0%) or high SII (>748.9; n = 178, 40.0%), and survival outcomes were compared between patients who did and did not receive pAC within each subgroup.

**Figure 3:**
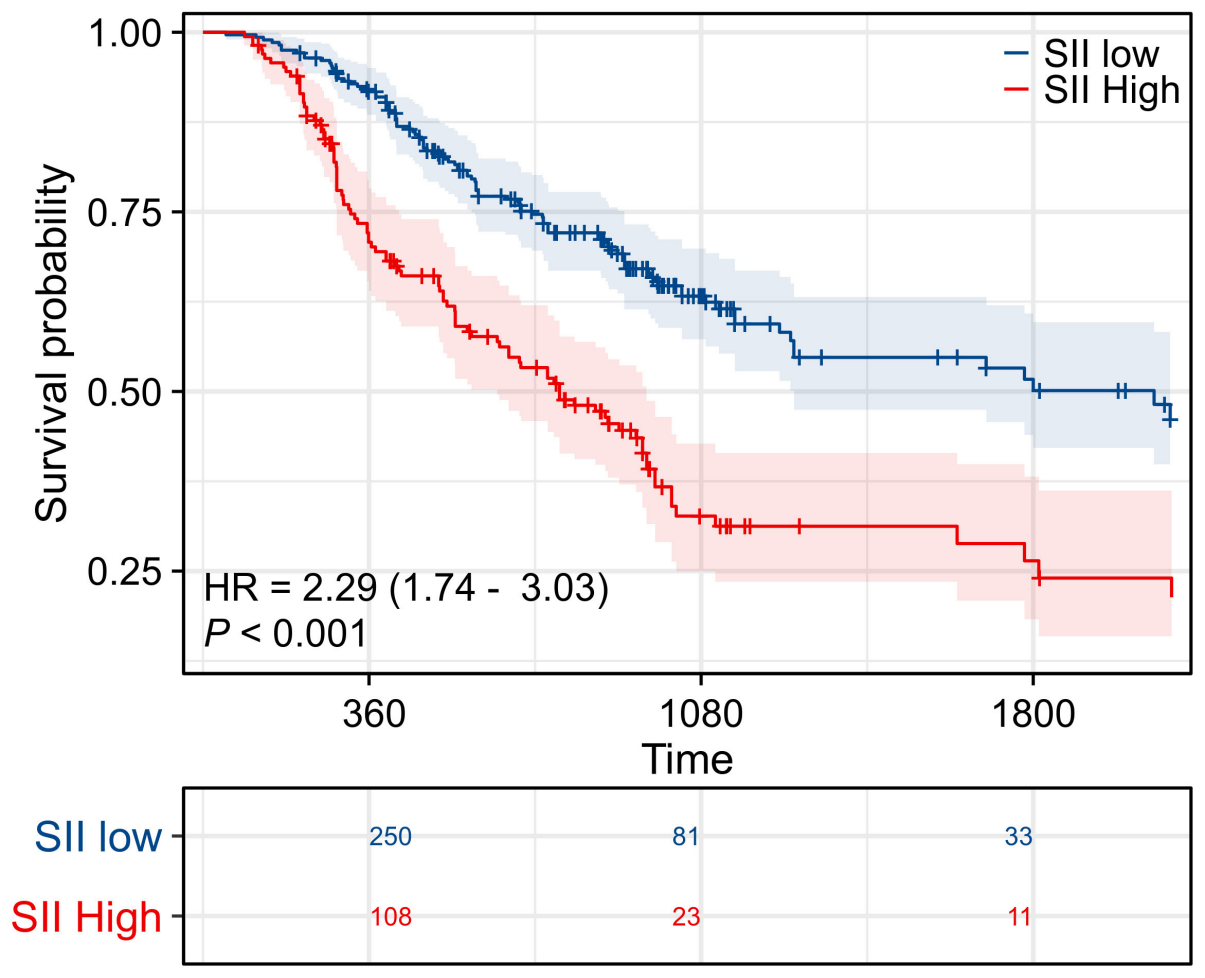
Prognostic significance of SII. Kaplan–Meier survival curves evaluating overall survival (OS) in patients stratified by systemic immune-inflammation index (SII). Patients in the high SII group demonstrated significantly worse survival compared with those in the low SII group, highlighting SII as an independent prognostic indicator.

**Figure 4:**
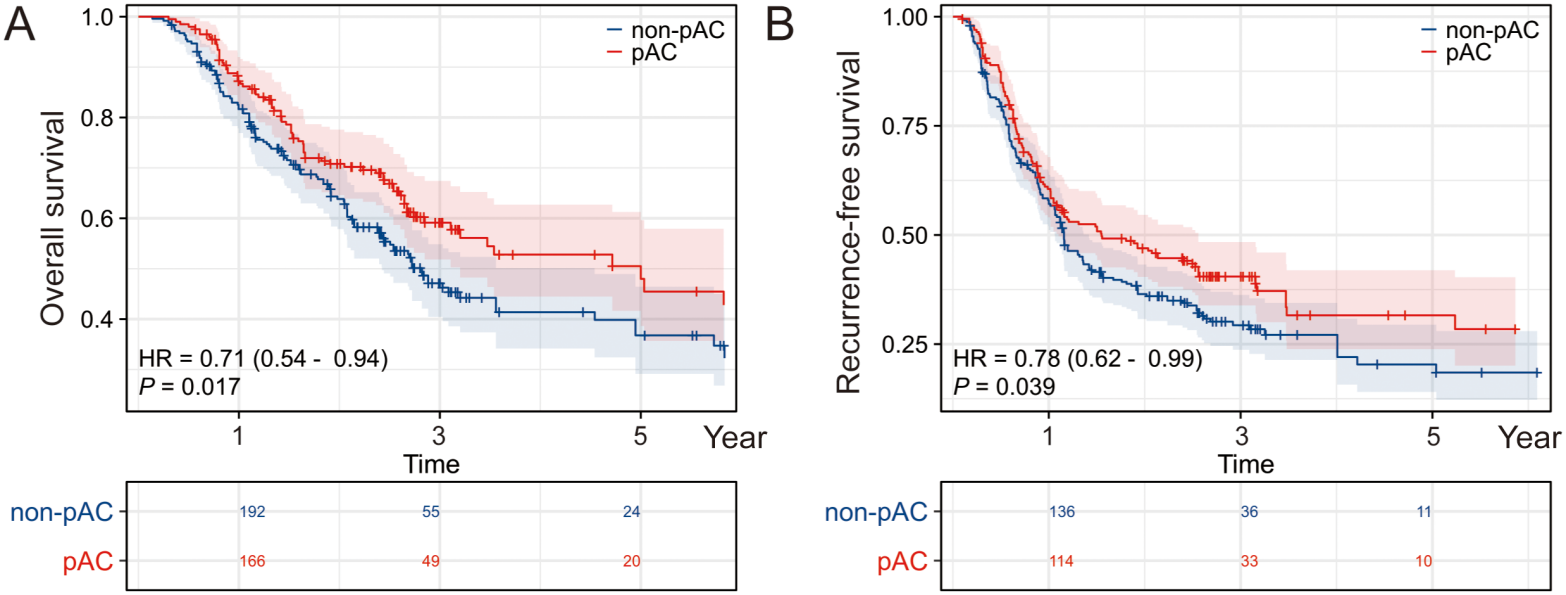
Prognostic effect of postoperative adjuvant chemotherapy stratified by SII level. Kaplan–Meier survival analyses comparing overall survival (OS) between patients who did or did not receive postoperative adjuvant chemotherapy (pAC), further stratified by systemic immune-inflammation index (SII) status. **(A)** In the high SII subgroup, OS did not significantly differ between pAC-treated and untreated patients. **(B)** In the low SII subgroup, patients who received pAC exhibited a notable survival advantage, indicating that SII may serve as a predictive biomarker for the benefit of pAC.

**Figure 3** showed the survival curves stratified by pAC in the low-SII and high-SII groups, respectively. Among low-SII patients (SII ≤ 748.9), receipt of adjuvant chemotherapy was not associated with a statistically significant survival improvement. Their Kaplan-Meier OS curves were nearly superimposable. Five-year OS was 49% for those who received pAC versus 55% for those who did not (difference not significant, log-rank p = 0.089; HR 0.71, 95% CI 0.48–1.05). In other words, ICC patients with low inflammatory index had relatively favorable long-term survival after surgery alone (around half were alive at 5 years), and adding chemotherapy did not appreciably change their outcomes. In contrast, among high-SII patients (SII > 748.9), we observed a clear benefit from adjuvant chemotherapy. High-SII patients who received pAC had a 5-year OS of ~33%, compared to only 23% of those who did not receive pAC. This 10% absolute increase translated to a significant difference (log-rank p = 0.022) with an adjusted HR of 0.62 (95% CI 0.42–0.94) favoring adjuvant therapy. The divergence of the curves in the high-SII group indicates a meaningful survival prolongation with chemotherapy that is absent in the low-SII group (**Figures 4A, B**).

Overall, our results indicate that SII is a strong prognostic indicator in ICC and a predictive biomarker of adjuvant chemotherapy benefit. High SII identifies patients with aggressive disease who are likely to benefit substantially from pAC, whereas low SII patients have good outcomes with surgery alone and can potentially be spared additional chemotherapy.

## 4 Discussion

In this retrospective study of a large ICC cohort, we found that SII is an independent prognostic factor and may serve as a key biomarker to guide postoperative therapy decisions. To our knowledge, this is the first study demonstrating that SII can stratify ICC patients with respect to the survival benefit of adjuvant chemotherapy. Our results confirmed that a heightened inflammatory state, as quantified by SII, portends more aggressive tumor behavior and poorer survival. Importantly, we showed for the first time that SII may help identify the subset of ICC patients who derive the greatest benefit from adjuvant chemotherapy. Patients with high SII had significantly improved long-term survival when treated with pAC, whereas those with low SII did not experience a meaningful benefit from chemotherapy. These findings support an approach of risk-adapted adjuvant therapy in ICC, tailoring treatment intensity to the patient’s inflammatory risk profile.

Firstly, our study corroborates that SII is an independent prognostic indicator in ICC, predictive of both overall and recurrence-free survival. SII is readily obtained from routine blood counts and reflects the complex interaction between tumor and host immunity. Mechanistically, an elevated SII indicates a relative neutrophilia and thrombocytosis with lymphocytopenia. Neutrophils and platelets can promote tumor progression by secreting pro-inflammatory cytokines, growth factors, and by suppressing anti-tumor immune effector cells, while lymphocytes (particularly cytotoxic T cells and NK cells) are central to anti-tumor immunity (33–36). Thus, a high SII suggests the patient’s immune microenvironment is skewed toward tumor-promoting inflammation and inadequate anti-tumor response. Prior studies for other subtypes of cholangiocarcinoma (hilar cholangiocarcinoma and distal cholangiocarcinoma) have observed that a high SII is associated with adverse outcomes (37–40). Our results extend this knowledge to ICC. Even after adjusting for tumor stage and other factors, SII remained a strong predictor of survival, implying that it captures biological aspects of the tumor-host interaction not fully accounted for by traditional staging (for example, the degree of tumor-induced systemic inflammation or immunosuppression). Clinically, SII is an attractive biomarker because it is inexpensive and easily obtained pre- and post-operatively, allowing dynamic risk stratification. Our data suggested that ICC patients with markedly elevated SII should be recognized as a high-risk group for recurrence and mortality. Such patients might benefit from more intensive postoperative surveillance and consideration of adjuvant therapies. Furthermore, SII is potentially a modifiable factor – emerging evidence indicates that perioperative inflammatory status can influence cancer outcomes (23). For instance, studies have suggested that controlling the postoperative inflammatory response or using immunomodulatory strategies might improve oncologic outcomes (41). Whether interventions to reduce systemic inflammation (e.g. NSAIDs, cytokine inhibitors, or immune-nutrition) can favorably impact recurrence in high-SII patients is an intriguing question for future research.

Secondly, although we evaluated LNR and found it valuable prognostically, LNR did not emerge as a predictor of differential chemotherapy benefit in our analysis. We confirmed prior observations that LNR is superior to the simple N stage in prognostication for ICC (42). In our data, once LNR was included, the conventional N0/N1 categorization lost significance, indicating LNR provides a more nuanced assessment of nodal disease burden. This echoes findings in colorectal and gastric cancers that the ratio of positive nodes improve prognostic accuracy compared to positive node count alone (43, 44). We observed that higher LNR was associated with incrementally worse survival, emphasizing the importance of thorough lymphadenectomy both for accurate staging and potentially for improving outcomes (by clearing disease). Our results underscore that surgeons should aim to dissect and examine an adequate number of lymph nodes (at least 6–8 nodes as some studies suggest) during ICC resection. This not only maximizes clearance of micro-metastatic disease but also enables precise calculation of LNR, which can guide postoperative risk stratification and treatment decisions.

One of the most clinically significant findings of our study is that SII can identify the ICC patient subgroups who benefit the most from adjuvant chemotherapy, and conversely those who may not require it. This finding is in line with recent research by Kawashima et al. and others, who reported that adjuvant therapy is not uniformly beneficial for all ICC patients, but confers particular benefit in high-risk subsets. In our cohort, high-SII patients who received adjuvant chemotherapy achieved about a 10% absolute improvement in 5-year survival compared to those who did not – an appreciable gain in the context of ICC. On the other hand, low-SII patients had relatively favorable survival (~50% at 5 years) with surgery alone; adding chemotherapy provided no significant survival advantage. This suggests that not all ICC patients require routine adjuvant chemotherapy – a substantial fraction of “low-risk” patients may achieve good long-term outcomes with surgery alone. By identifying these patients (using SII and possibly other markers), we can avoid subjecting them to the unnecessary toxicity, cost, and morbidity of chemotherapy that is unlikely to help them. From a practical standpoint, we propose incorporating SII into postoperative risk assessment for ICC. Patients with high SII (and/or other high-risk features) would be strong candidates for adjuvant therapy, whereas those with low SII and otherwise low-risk profiles might be observed after resection without chemotherapy.

It is noteworthy that our multivariate analysis confirmed adjuvant chemotherapy itself as an independent factor improving ICC survival (adjusted HR ~0.63), consistent with several recent retrospective studies and meta-analyses. For instance, a contemporary meta-analysis reported that adjuvant therapy significantly prolongs OS in resected ICC, especially in patients with nodal metastases or positive margins. Altman et al. conducted a multi-institutional study showing a trend toward improved survival with adjuvant chemotherapy, supporting its consideration in most ICC patients with curative resection. However, these studies did not specifically distinguish which patients might not need chemotherapy. Our study adds to the literature by suggesting that perhaps ~20–40% of ICC patients (those we classified as “low-risk” by SII and other factors) could forego routine adjuvant chemotherapy without compromising their prognosis. If validated prospectively, this could have significant clinical implications – reducing overtreatment and focusing resources and therapy on patients most likely to benefit.

Besides SII, our study reaffirmed the prognostic importance of several known factors in ICC. We observed that microvascular invasion was a powerful predictor of early recurrence and death, aligning with innumerable studies identifying vascular invasion as a hallmark of aggressive tumor biology. Similarly, larger tumor size and multifocality (together reflecting overall tumor burden) mainly affected recurrence risk in our data, consistent with the concept of the Tumor Burden Score (TBS) as a prognostic metric in ICC. Interestingly, tumor differentiation and perineural invasion were associated with outcomes on univariate analysis but did not remain in the multivariate model, likely due to collinearity with other factors (e.g. poorly differentiated tumors often coincide with high SII or vascular invasion, such that their effect is captured by the latter). We also found that although CA19–9 is the most used tumor marker in ICC, an elevated CEA level was an independent adverse prognostic factor for RFS (HR ~1.5, p = 0.016). This is in line with some reports that ICC patients with elevated CEA have worse outcomes, even though CEA is traditionally associated more with colorectal malignancies. Our data suggest that ICC patients with an abnormal preoperative CEA should be considered higher risk for recurrence, underscoring that clinicians should pay attention to CEA as well as CA19–9 in ICC management.

We acknowledge several limitations of this study. First, as a retrospective single-center analysis, selection biases are inherent. The decision to administer adjuvant chemotherapy was not randomized but based on clinical factors and physician judgment, which could confound survival comparisons. We attempted to mitigate this by comparing baseline characteristics and performing multivariable adjustments; notably, many high-risk patients did get chemotherapy, as intended. However, it is impossible to eliminate unmeasured confounders in such an analysis. Therefore, any observed “adjuvant chemotherapy benefit” should be interpreted with caution, recognizing potential biases. Ideally, a prospective randomized trial is needed to confirm which ICC patients truly benefit from adjuvant therapy. However, conducting such trials is challenging due to the relative rarity of ICC – for example, the phase III trials to date had to include all biliary tract cancers to accrue enough patients. In the absence of large ICC-specific RCTs, our retrospective findings offer valuable guidance to clinicians. Second, the SII cutoff value determined by our study (≈749) may not be universally applicable. This threshold was derived via a MaxStat algorithm on our dataset and might differ in other cohorts. Prior studies in other cancers have used SII cutoffs of ~600 or 800. Therefore, larger multi-center data would be useful to validate the optimal SII cutoff for ICC prognostication. Third, we only evaluated SII at a single preoperative time point. It is conceivable that the dynamic change in SII after tumor resection could have prognostic value – for instance, if SII remains high or quickly rebounds postoperatively, might that predict early recurrence? We did not analyze longitudinal post-surgery SII trends, which is an area for future research. Additionally, we chose to focus on SII and did not include some other inflammatory indices like NLR or PLR in multivariate models, because SII incorporates information from these components (neutrophils, platelets, lymphocytes). It is possible that combining multiple immune markers or integrating them with emerging biomarkers (e.g. circulating tumor DNA, molecular profiling) could yield even more robust predictive models. Developing a composite prognostic score that blends host inflammation markers with tumor-specific genetic features might further refine individualized risk prediction and decision-making for adjuvant therapy – a promising direction for future investigation.

Despite these limitations, our study provides clear practical implications for post-surgical management of ICC. We demonstrate that a one-size-fits-all approach to adjuvant therapy is suboptimal; instead, personalized, risk-stratified treatment is warranted in ICC following resection. In essence, we emphasize moving from an “all patients get chemotherapy” paradigm to one guided by objective risk metrics. Similar personalized strategies are already being explored in other malignancies. For example, in colorectal cancer, ongoing trials aim to use molecular assays and inflammatory scores to select which stage II patients truly need chemotherapy. In esophageal cancer, as mentioned, LNR is being used to tailor postoperative therapy recommendations. We propose that SII (along with tumor burden and nodal status) be considered for integration into postoperative ICC management algorithms. Patients with high-risk inflammatory profiles (high SII) should receive adjuvant chemotherapy because they stand to gain significantly (as our data showed ~10% absolute survival benefit at 5 years). Conversely, low-SII patients – especially if they also have no nodal metastasis and other favorable features – may be observed after surgery with vigilant follow-up, reserving chemotherapy for salvage if needed. This approach would allocate treatment resources more rationally: intensifying treatment for those who truly need it and avoiding over-treatment in those who likely do not. The potential benefits include improved overall survival for the population (by effectively treating high-risk patients) and better quality of life for low-risk patients (by sparing them unnecessary side effects).

## 5 Conclusion

Preoperative SII is a strong prognostic biomarker in ICC, identifying patients at high risk of recurrence and mortality. High-SII patients benefit significantly from adjuvant chemotherapy, whereas low-SII patients have favorable outcomes with surgery alone and may avoid unnecessary treatment. Incorporating SII into postoperative risk stratification allows personalized therapy, optimizing survival while minimizing overtreatment.

## Data Availability

The original contributions presented in the study are included in the article/**Supplementary Material**. Further inquiries can be directed to the corresponding authors.
