# Supplementary material for: Systemic immune-inflammation index: a key biomarker guiding personalized adjuvant chemotherapy in intrahepatic cholangiocarcinoma
Source: Front Oncol. 2025 Oct 31;15:1702336. doi: 10.3389/fonc.2025.1702336 (PMC12615207; doi:10.3389/fonc.2025.1702336)
Supplement: Supplementary file 1 [file Table1.docx]

Supplementary Material

# Supplementary Tables 1. Univariate and multivariate Cox regression analyses of RFS for ICC patients

| **Characteristics** | **Total (N)** | **Univariate analysis** |  |  | **Multivariate analysis** |  |
| --- | --- | --- | --- | --- | --- | --- |
|  |  | **Hazard ratio (95% CI)** | **P value** |  | **Hazard ratio (95% CI)** | **P value** |
| **Gender** | 445 |  |  |  |  |  |
| Female | 180 | Reference |  |  |  |  |
| Male | 265 | 1.158 (0.915 - 1.466) | 0.222 |  |  |  |
| **Initial treatment age** | 445 | 0.996 (0.985 - 1.008) | 0.556 |  |  |  |
| **Differentiation** | 445 |  |  |  |  |  |
| Moderate | 174 | Reference |  |  |  |  |
| Poor and Moderate-Poor | 244 | 1.103 (0.868 - 1.402) | 0.423 |  |  |  |
| Moderate-Well and Well | 27 | 0.721 (0.414 - 1.255) | 0.247 |  |  |  |
| **TNM stage** | 445 |  |  |  |  |  |
| Stage II | 233 | Reference |  |  | Reference |  |
| Stage III | 212 | 2.245 (1.774 - 2.840) | < 0.001 |  | 1.677 (1.123 - 2.505) | 0.011 |
| **Pathological T stage** | 445 |  |  |  |  |  |
| T2 | 295 | Reference |  |  | Reference |  |
| T3 | 139 | 1.696 (1.332 - 2.159) | < 0.001 |  | 0.951 (0.672 - 1.346) | 0.777 |
| T4 | 11 | 2.044 (1.005 - 4.159) | 0.048 |  | 1.058 (0.495 - 2.261) | 0.884 |
| **Pathological N stage** | 445 |  |  |  |  |  |
| N0 | 307 | Reference |  |  | Reference |  |
| N1 | 138 | 2.192 (1.721 - 2.792) | < 0.001 |  | 1.004 (0.647 - 1.557) | 0.987 |
| **CA 19-9** | 445 | 1.000 (1.000 - 1.000) | < 0.001 |  | 1.000 (1.000 - 1.000) | 0.147 |
| **CEA** | 445 | 1.009 (1.005 - 1.012) | < 0.001 |  | 1.005 (1.001 - 1.010) | 0.016 |
| **Diameter of tumor** | 445 | 1.093 (1.038 - 1.150) | < 0.001 |  | 1.077 (1.016 - 1.142) | 0.013 |
| **Postoperative adjuvant chemotherapy** | 445 |  |  |  |  |  |
| No | 245 | Reference |  |  | Reference |  |
| Yes | 200 | 0.781 (0.618 - 0.987) | 0.039 |  | 0.666 (0.519 - 0.855) | 0.001 |
| **Tumor position** | 445 |  |  |  |  |  |
| Left liver lobe | 194 | Reference |  |  |  |  |
| Right liver lobe | 188 | 1.050 (0.818 - 1.348) | 0.702 |  |  |  |
| Middle liver lobe | 63 | 1.019 (0.720 - 1.442) | 0.917 |  |  |  |
| **Liver capsule invasion** | 445 |  |  |  |  |  |
| Yes | 299 | Reference |  |  | Reference |  |
| No | 146 | 0.736 (0.568 - 0.954) | 0.020 |  | 0.920 (0.700 - 1.209) | 0.547 |
| **Satellite nodules** | 445 |  |  |  |  |  |
| No | 387 | Reference |  |  |  |  |
| Yes | 58 | 1.341 (0.966 - 1.861) | 0.079 |  |  |  |
| **Intravascular carcinoma embolus** | 445 |  |  |  |  |  |
| Yes | 202 | Reference |  |  | Reference |  |
| No | 243 | 0.640 (0.508 - 0.806) | < 0.001 |  | 0.729 (0.569 - 0.935) | 0.013 |
| **Perineural invasion** | 445 |  |  |  |  |  |
| Yes | 200 | Reference |  |  | Reference |  |
| No | 245 | 0.763 (0.605 - 0.962) | 0.022 |  | 0.994 (0.766 - 1.289) | 0.961 |
| **Surrounding tissues Invasion** | 445 |  |  |  |  |  |
| Yes | 88 | Reference |  |  |  |  |
| No | 357 | 0.767 (0.579 - 1.016) | 0.064 |  |  |  |
| **Number of lymph node dissections** | 445 | 1.010 (0.993 - 1.027) | 0.246 |  |  |  |
| **Proportion of positive lymph nodes** | 442 | 4.342 (2.930 - 6.433) | < 0.001 |  | 2.212 (1.007 - 4.859) | 0.048 |
| **Operation time** | 445 | 1.002 (1.000 - 1.003) | 0.037 |  | 1.000 (0.999 - 1.002) | 0.557 |
| **Intraoperative hemorrhage** | 445 | 1.000 (1.000 - 1.001) | 0.003 |  | 1.000 (1.000 - 1.000) | 0.175 |
| **Intraoperative blood transfusion** | 445 |  |  |  |  |  |
| Yes | 67 | Reference |  |  | Reference |  |
| No | 378 | 0.682 (0.503 - 0.926) | 0.014 |  | 0.704 (0.506 - 0.981) | 0.038 |
| **Postoperative days** | 445 | 1.036 (1.013 - 1.059) | 0.002 |  | 1.012 (0.989 - 1.035) | 0.322 |
| **SII** | 445 | 1.001 (1.001 - 1.001) | < 0.001 |  | 1.001 (1.001 - 1.001) | < 0.001 |
| **CALLY** | 445 | 0.999 (0.993 - 1.005) | 0.658 |  |  |  |
| **PNI** | 445 | 1.001 (0.994 - 1.007) | 0.835 |  |  |  |
